# Mitochondrial Dysfunction may explain symptom variation in Phelan-McDermid Syndrome

**DOI:** 10.1038/srep19544

**Published:** 2016-01-29

**Authors:** Richard E. Frye, Devin Cox, John Slattery, Marie Tippett, Stephen Kahler, Doreen Granpeesheh, Shirish Damle, Agustin Legido, Michael J. Goldenthal

**Affiliations:** 1 University of Arkansas for Medical Sciences, Department of Pediatrics, Arkansas Children’s Hospital Research Institute, Little Rock, Arkansas, AR 72202, USA; 2 Kansas University Medical Center, Kansas City, Kansas, KS, USA; 3 Center for Autism and Related Disorders, Inc., Woodland Hills, California, CA, USA; 4 Drexel University College of Medicine, Department of Pediatrics, Neurology Section, St. Christopher’s Hospital for Children, Philadelphia, PA 19134, USA

## Abstract

Phelan-McDermid Syndrome (PMS), which is defined by a deletion within 22q13, demonstrates significant phenotypic variation. Given that six mitochondrial genes are located within 22q13, including complex I and IV genes, we hypothesize that mitochondrial complex activity abnormalities may explain phenotypic variation in PMS symptoms. Complex I, II, II + III and IV activity was measured in 51 PMS participants. Caretakers completed questionnaires and provided genetic information through the PMS foundation registry. Complex activity was abnormal in 59% of PMS participants. Abnormalities were found in complex I and IV but not complex II + III and II activity, consistent with disruption of genes within the 22q13 region. However, complex activity abnormalities were not related to specific gene deletions suggesting a “neighboring effect” of regional deletions on adjacent gene expression. A specific combination of symptoms (autism spectrum disorder, developmental regression, failure-to-thrive, exercise intolerance/fatigue) was associated with complex activity abnormalities. 64% of 106 individuals in the PMS foundation registry who did not have complex activity measured also endorsed this pattern of symptoms. These data suggest that mitochondrial abnormalities, specifically abnormalities in complex I and IV activity, may explain some phenotypic variation in PMS individuals. These results point to novel pathophysiology mechanisms and treatment targets for PMS patients.

Phelan-McDermid Syndrome (PMS) is a genetic syndrome defined by a deletion in the 22q13 chromosomal region and a variety of clinical findings including physical dysmorphology, medical conditions such as seizures, both growth failure and overgrowth, hypotonia, gastrointestinal disturbances and neurodevelopmental disorders such as developmental delays and autism spectrum disorder (ASD)^1^. The association between PMS and ASD has raised particular interest in the SHANK3 gene, a gene which lies in the distal end of the 22q13 region and is important in synaptic function^2^. However, individuals with PMS have variations in their development, behavior and medical characteristics that cannot be fully explained by the SHANK3 deletion. Furthermore, children without a SHANK3 deletion can demonstrate neurodevelopmental disorders suggesting that other genes within this region may have a role in explaining symptoms in individuals with PMS^3^.

Recently, Frye^4^ noted that 6 mitochondrial genes are proximal to the SHANK3 gene within the 22q13 region, suggesting that copy number variations in this chromosomal region may have the potential to cause abnormal mitochondrial function. These genes include those essential for complex I (NDUFA6) and IV (SCO2) function of the electron transport chain (ETC) as well as mitochondrial DNA (TYMP), RNA (TRMU) and fatty acid (CPT1B) metabolism and tricaboxylic acid cycle function (ACO2)^5^,^6^,^7^,^8^,^9^,^10^,^11^,^12^,^13^,^14^,^15^,^16^,^17^,^18^. Table 1 outlines these genes as well as the mitochondrial diseases with which they have been associated.

Since most PMS individuals have a deletion that includes at least one of these genes, these mitochondrial genes are likely affected in many PMS individuals. As individuals with mitochondrial abnormalities can have many of the same medical and behavioral abnormalities commonly reported in PMS individuals, it is very possible that abnormalities in mitochondrial function could account for some of the variation in symptoms associated with PMS.

Thus, we hypothesize that a significant percent of PMS individuals have mitochondrial abnormalities and that such abnormalities account for variation in PMS symptoms. We measured activity of ETC Complex I, II, III and IV using a validated buccal swab procedure^19^. Since genes for Complex I and IV, but not II or III, are located in 22q13, we hypothesized that Complex I and IV but not II or III would show abnormalities. We considered both underactivity and overactivity of ETC complexes since PMS is associated with ASD and mitochondrial overactivity has been associated with ASD in several studies^20^,^21^,^22^,^23^,^24^,^25^,^26^. We also determined whether patients with abnormal mitochondrial complex activity demonstrate symptoms of classic mitochondrial disease, such as developmental regression, failure to thrive, exercise intolerance, fatigue and autism spectrum disorder. Lastly, we investigated whether a genotype-phenotype correspondence exists. While deletions of genes for complex I and IV could account for ETC activity abnormalities, the “neighboring effect” can result in dysfunction of neighboring genes even if their genetic material is not specifically deleted^27^,^28^,^29^,^30^,^31^,^32^,^33^,^34^. It should be noted that an extensive mitochondrial workup was not completed on participants as part of this study. Thus, a diagnosis of mitochondrial disease cannot be provided and, rather, participants with one or more ETC complex activities outside of the normal range are referred to as having mitochondrial abnormalities or complex activity abnormalities.

## Results

### Mitochondrial Function in Phelan-McDermid Syndrome Patients

**Figure 1:**
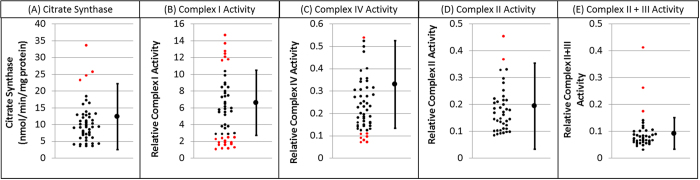
Activity of Citrate Synthase and Complex I, II, II + III and IV for children with Phelan-McDermid Syndrome. As predicted by the genes commonly affected by this syndrome, activity of ETC Complex I and IV is abnormal in many cases. For each graph the control mean and minimal and maximum limits of normal are provided on the right. Individual participant data is provided on the left of each graph with individuals that have values outside of the normal range highlighted in red.

Table 2 outlines the results of the statistical analysis. Overall 59% (30/51) of PMS individuals demonstrated ETC complex activity outside the control range (Fig. 1, Table 2). Complex I activity was outside of the control range for 43% (22/51), which is significantly more than expected by chance alone. Complex I activity variance was significantly greater in PMS participants as compared to the control group but mean Complex I activity for PMS participants was not different than the control group. Complex IV activity was outside the normal range for 16% (8/51), which is significant more than expected by chance alone. Mean Complex IV activity was significantly lower in PMS participants as compared to the control group. Although Complex II + III activity variability was significantly greater for PMS participants as compared to the control group, Complex II and Complex II + III activity was not significantly abnormal in more PMS participants than expected by chance alone and mean activity was not significantly difference between PMS participants and the control group.

**Figure 2:**
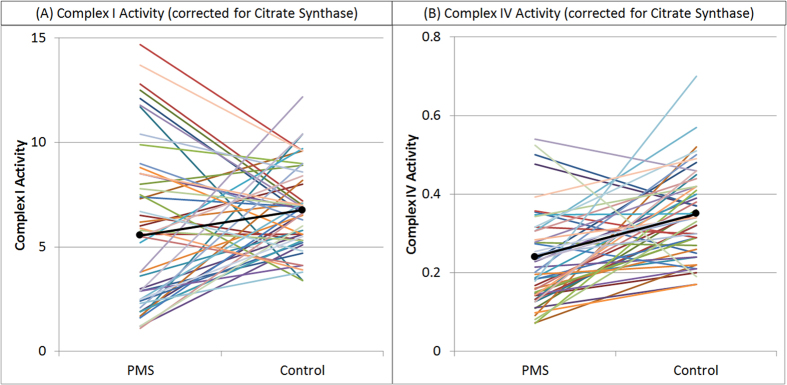
Comparison of ETC Complex I and Complex IV activity between Phelan-McDermid Syndrome participants and age-matched controls. Complex IV activity is significantly lower in the Phelan-McDermid Syndrome participants as compared to the age-matched controls. Although Complex I activity is also significantly lower in the Phelan-McDermid Syndrome participants, the variability in Complex I activity is significantly greater for the Phelan-McDermid Syndrome participants as compared to the age-matched controls. These findings confirm the findings of the previous analysis that suggests abnormal activity of both Complex I and IV in Phelan-McDermid Syndrome participants.

To confirm that the above results were not due to differences in age of the PMS and control groups, complex I and IV activity was compared between PMS participants and 51 age-matched controls (Fig. 2). PMS participants demonstrated significantly lower Complex I activity [t(50) = 2.10, p = 0.01] and significantly greater variation [F(50,50) = 3.20, p < 0.0001] as compared to the age-matched controls [Mean (SD): PMS 5.55 (3.67), Controls 6.76 (2.05)]. PMS participants demonstrated significantly lower Complex IV activity [t(50) = 4.81, p < 0.0001] than age-matched controls [Mean (SD): PMS 0.24 (0.12); Controls 0.35 (0.11)] but the variance was not significantly different between groups. Neither the mean nor variance in Citrate Synthase was significantly different between the PMS participants and age-matched controls. These findings confirm differences in Complex I and Complex IV activity between PMS participants and controls found in the previous analysis.

Table 3 outlines the overlap of complex function abnormalities within the PMS participants. Overall, most, 77% (24/31), of the PMS participants with ETC complex abnormalities had only one ETC complex affected. Complex I and IV abnormalities were isolated a majority of the time while Complex II abnormalities were always isolated. Abnormalities in Complex II + III (elevated activity) were always associated with a Complex I abnormality (depressed activity in 2 cases and elevated activity in 1 case) and abnormalities in Citrate Synthase (3 elevated activity and 1 depressed activity) were usually associated with a Complex IV abnormality (depressed activity in all cases).

### Mitochondrial Complex Abnormalities and Autism Symptoms

61% (19/31) of PMS participants reported symptoms consistent with ASD. Although individuals with complex activity abnormalities were not significantly more likely to report symptoms consistent with the ASD diagnosis, more ASD communication symptoms were reported by participants that demonstrated complex activity abnormalities [t(30) = 2.34, p = 0.03; Table 4].

### Mitochondrial Abnormalities and Mitochondrial Genes

Table 5 outlines the distribution of deleted regions for all PMS participants as well as those with and without mitochondrial complex activity abnormalities. The deleted region did not include ACO2 or NDUF6 for any case. 37% (10/27) of PMS participants demonstrated a deletion of the TRMU gene. Because of the proximity between SCO2, TYMP and CPT1B, all 85% (23/27) participants that had deletions in this region had all three genes deleted. Mitochondrial ETC complex activity abnormalities were not more prevalent in PMS participants with and without deletions in any of these regions.

### Mitochondrial Function and Medical Conditions, Symptoms and Treatments

Several symptoms commonly associated with classic mitochondrial disease (MD) were more prevalent in participants identified with ETC complex activity abnormalities, including feeding problems, failure to thrive, exercise intolerance, and fatigue (Table 6). Attention problems (p = 0.03) and oppositional defiant disorder (p = 0.05) were more prevalent in the PMS participants without ETC complex activity abnormalities (Table 6).

Sleep quality was similarly abnormal in individuals with and without ETC complex activity abnormalities (Table 6). However, the number of minutes slept per night on the weekends, but not the weekdays, was significantly greater for those with ETC complex activity abnormalities [t(29) = 3.20, p < 0.01].

There was a low rate of alternative therapies use, expect for probiotics, with little difference between those with normal and abnormal ETC complex activity, providing no support for the idea that ETC complex activity abnormalities could have been due to dietary or vitamin therapies (Table 6).

### Symptoms Specific for Mitochondrial Dysfunction

We investigated whether symptoms common to classically defined MD, included regression, failure to thrive, exercise intolerance, and fatigue, could identify PMS individuals with ETC complex activity abnormalities. Exercise intolerance and fatigue were combined into one variable since these symptoms are very similar. We included ASD in the list of MD symptoms since ASD has been associated with classically defined MD. The forward stepwise logistic regression selected the interaction between Exercise Intolerance/Fatigue by ASD in addition to the basic symptoms. Thus, having symptoms of ASD, developmental regression, failure to thrive and/or exercise intolerance/fatigue was associated with a greater likelihood of abnormal ETC complex activity while having both ASD and exercise intolerance/fatigue reduced the likelihood of having ETC complex activity abnormalities.

The discriminant analysis using these symptom had a Canonical Correlation of 0.67 (Likelihood Ratio = 0.54, F(5,24) = 4.00, p < 0.01) and a discriminant function with an accuracy of 87% (Sensitivity 88%, Specificity 85%). Cross-validation showed similar results (Accuracy 83%, Sensitivity 88%, Specificity 79%). The two false positive individuals demonstrated all of the characteristics found to identify individuals with ETC complex activity abnormalities. The discriminant function was applied to questionnaires from 106 additional patients from the PMS registry. This calculation predicted that 64% of the 106 registry individuals had symptoms consistent with ETC complex activity abnormalities. This is very similar to the 59% found to have ETC complex activity abnormalities using direct testing to ETC function with buccal swabs in our participant sample.

### Comparison of Mitochondrial Subgroups

We compared clinical features in those with complex I underactivity to those with complex I overactivity (Table 7). Individuals with PMS and complex I overactivity had a higher rate of ASD and a pattern of regression that was more characteristic of ASD. Specifically, regression in these individuals was characterized by a primarily loss of social skills within 12–24 months of age. PMS individuals with complex I underactivity had characteristics more typical of classical defined MD, including constipation, abdominal pain, failure to thrive, short stature, exercise intolerance, fatigue, weakness, seizures, regression in motor skills, regression association with triggers, multiple regressions and 1^st^ regression occurring outside the typical age for ASD.

## Discussion

In this study we demonstrated that 59% of individuals with PMS had abnormalities in ETC complex activity, thereby supporting our hypothesis that disruption of mitochondrial genes in the 22q13 region may disrupt mitochondrial function. We predicted that Complex I and Complex IV abnormalities would be more common than expected in PMS individuals while abnormalities in Complex II and II + III would not. We confirmed this hypothesis by demonstrating that 43% and 16% of PMS individuals demonstrated Complex I and IV activity outside the control range, respectively, which is significantly more than expected. In contrast, only 8% and 5% of individuals with PMS demonstrated ETC Complex II + III and II activity outside the range of normal, which is not more than expected.

Abnormalities in ETC Complex I activity was most common with abnormal elevation in Complex I activity in 14% and abnormal depression in Complex I activity in 29%. This resulted in a greater variation in ETC Complex I activity in individuals with PMS as compared to control individuals. Although, none of the individuals with ETC Complex I abnormalities demonstrated a deletion in the NDUFA6 gene, studies have shown that genes adjacent to regions of copy number variation can be dysregulated despite being structurally intact^27^,^28^,^29^,^30^,^31^,^32^,^33^,^34^ with genes several megabases away being affected^29^,^33^. This “neighboring effect” could explain the lack of specific genotype-phenotype and why there was no specific association between abnormalities in ETC Complex I activity and a specific deletions of the NDUFA6 gene. Alternatively, intact genes within regions of large chromosomal deletions have been shown to have point mutation, resulting in expression of recessive mitochondrial disorders^10^. This suggests that the same pathological processes that caused the copy number variation may also cause smaller changes in nearby genes that would not be detected by microarray. Since we did not sequence the intact genes in and near the copy number variations, it is possible that point mutations in mitochondrial genes may have contributed to the ETC complex activity abnormalities.

Clinical characteristics of classic MD were common in PMS individuals who demonstrated ETC complex activity abnormalities and differentiated those PMS individuals with and without ETC complex activity abnormalities with 87% accuracy. In addition, we found that 64% of PMS individuals in the PMS foundation registry also endorsed symptoms consistent with ETC complex activity abnormalities. Interestingly two individuals without ETC complex activity abnormalities had many MD symptoms, raising the possibility that non-ETC mitochondrial genes, such as CPT1B, which can be associated with common MD symptoms, could have been affected in these individuals. Overall, these data suggest that there may be a subgroup of PMS individuals who manifest mitochondrial symptoms and may be at risk for having mitochondrial abnormalities. Given that there are treatments for mitochondrial disorders, this opens up a novel path for treatment of PMS individuals.

ETC Complex I overactivity has been reported in ASD^20^ and mitochondrial overactivity has been associated with ASD in muscle^21^,^22^, skin^23^, brain^24^ and cell lines^25^,^26^. Interestingly, the subgroup of PMS individuals with ETC Complex I overactivity had clinical characteristics that were more characteristic of ASD, particularly regression characterized by the primary loss of social skills within the age typical for regression in children with ASD (i.e., 12–24 months of age). In contrast, those with ETC Complex I underactivity demonstrated characteristic more consistent with classically defined MD, including constipation, failure to thrive, short stature, exercise intolerance, fatigue, weakness, seizures, regression characterized by loss of motor skills and multiple regressions with the age of 1^st^ regression occurring outside the typical age for children with ASD.

This study has limitations, including a lack of skin and/or muscle biopsies to confirm the buccal swab findings, the fact that only a subset of participants completed the registry questionnaires and that ETC Complex activity was not assessed simultaneously in controls. In addition, the genetic information was derived from clinical reports using techniques which vary in their resolution and sequencing of important genes responsible for mitochondrial function was not performed. However, the genetic findings for this PMS sample is similar to samples from other recent studies^35^,^36^.

Overall, this study provides important initial data to support the further investigation of abnormalities in mitochondrial function in individuals with PMS. Future studies should investigate a wider variety of mitochondrial markers in order to consider other mitochondrial abnormalities aside from ETC function (e.g, carnitine abnormalities) and consider verifying ETC abnormalities across multiple tissue types.

This study suggests that mitochondrial dysfunction, as measured by abnormal ETC complex activity, may affect a significant portion of individuals with PMS. Although further research is needed to confirm these findings, it would be wise for clinicians to have a high index-of-suspicion for mitochondrial abnormalities in individuals with PMS as identifying such individuals could lead to novel approaches that may improve certain symptoms.

## Methods

This study was approved by the Institutional Review Board at the University of Arkansas for Medical Sciences. The methods were carried out in accordance with the approved guidelines and protocol. Informed consent was obtained from at least one parent of each subject in accordance with the protocol approved by the Institutional Review Board at the University of Arkansas for Medical Sciences.

### Participants

51 individuals diagnosed with PMS (approximately 6.3% of the known cases) were recruited as part of the international biennial meeting of the PMS Foundation (Venice, FL). Participant age ranged from 2 to 34 years of age [mean (SD) 9.7 years (6.8 years)] with 27 (66%) being female.

Controls of similar age and gender included 106 healthy individuals without neurological disease who have been described and used in previous studies^19^. Controls ranged in age from 2 to 49 years of age [mean (SD) 10.2 years (8.4 years)] with 52 (49%) being female. From this set of controls, 51 were age matched to participant for the additional analysis. The means (SD) age of the age-matched subset was 9.9 (6.7) years and the mean (SD) difference in age between the matches was 0.1 (0.4) years with the maximum difference of 1 year.

In a previous report, it was found that there was no correlation between protein activities and age and no difference in protein activities across ethnicity or race in both controls and previously studied MD patients^19^.

### Mitochondrial Activity Measurement

The senior author (MJG) performed the buccal cell collection using Catch-All Buccal Collection Swabs (Epicentre Biotechnologies, Madison, WI) in a private room. Four swabs where collected by firmly pressing a swab against the inner cheek while twirling for 30 s. Swabs were clipped and placed in 1.5 ml microcentrifuge tubes that were labeled and placed on dry ice for overnight transportation to the senior author’s laboratory.

Buccal extracts were prepared using an ice-cold buffered solution (Buffer A, ABCAM) containing protease inhibitor cocktail and membrane solubilizing non-ionic detergent and cleared of insoluble cellular material by high speed centrifugation at 4 °C. Duplicate aliquots of the protein extract were analyzed for protein concentration using the bicinchoninic acid method (Pierce Biotechnology, Rockford, IL). Samples were typically stored at −80 °C for up to 1 week prior to enzymatic analysis.

Dipstick immunocapture assays measured ETC Complex I activity using 50 μg extracted protein^19^,^37^,^38^. Signals were quantified using a Hamamatsu immunochromato reader (MS 1000 Dipstick reader). Raw mABS (milliAbsorbance) results were corrected for protein concentration and data were expressed as percentages of the values obtained with control extracts run on the same assay. ETC Complex IV and Citrate Synthase (CS) activity were assessed using standard spectrophotometric procedures in 0.5 ml reaction volume.

ETC Complex II and Complex II + III activity was measured using extracts were prepared from 1–2 buccal swabs in ice-cold 300 ul STE buffer containing lauryl maltoside and a protease inhibitor cocktail and vortexed for 1 min. After centrifugation of the extract at 15,000 g for 8 min, the supernatant was used. Complex II and combined Complex II + III activities were spectrophotometrically determined in two separate succinate-driven reactions by measuring the ubiquinone-initiated oxidation of DCPIP as gauged by the decrease in absorbance at 600 nm, and with the reduction of cytochrome *c* as gauged by the increase in absorbance at 550 nm, respectively^39^.

Specific activities of respiratory complexes were initially expressed as nanomoles per mg protein per minute and normalized to CS activity levels. The use of activity ratios is well established and provides a much narrower range of control values as compared to activities expressed on the basis of protein content.

### PMS Foundation Registry Data Collection

The PMS Foundation registry is an information repository for PMS patients. All participants in whom a buccal swab was collected had an entry in the PMS foundation registry with basic demographic information.

Individual genetic reports were uploaded to the registry and curated by a genetic counselor as part of the PMS foundation registry. This information includes the start and end point of the deletion as well as the length of the deletion, the genomic build and karyotype. The information was reviewed by a genetic counselor in training (DC, 2^nd^ author) under the supervision of a very experienced geneticist (SK, 5^th^ author) for the purpose of this paper. This genetic information was original reviewed by DC (2^nd^ author) as part of her master’s thesis under the supervision of several experts in genetics, statistics and medicine.

The first author (REF) designed versions of non-copyrighted behavioral questionnaires and customized medical history questionnaires that were integrated into the registry. These surveys queried: alternative medical treatments, ASD symptoms, general health symptoms and disorders, behavioral symptomatology, symptoms of regression, and sleep habits. To obtain questionnaire information, a letter of invitation was emailed to all individuals in the registry asking then to log into the registry and complete the questionnaires. They could complete one or all of questionnaires at one time.

ASD symptoms were assessed using the Autism Symptoms Questionnaire (ASQ) which is a DSM-IV-TR based checklist which has been used to rate ASD symptoms in previous studies^40^,^41^. Sleep symptoms were assessed using the Children’s Sleep Habits Questionnaire, a validated questionnaire^42^ that has been used in children with neurodevelopmental disabilities^43^. The Vanderbilt Diagnostic Rating Scale, a widely used scale that has been adopted by the American Academy of Pediatrics^44^ was used to detect common behavioral abnormalities. A medical questionnaire asked about clinical features associated with PMS and/or mitochondrial disease. A regression questionnaire queried whether or not regression occurred, how many times regression occurred, age of the first and last regression as well as whether the regression included a loss of language, social, fine motor or gross motor skills, loss of other skills and whether there were any associated factors or trigger that temporally preceded the regression. Finally, an alternative medication and treatment questionnaire asked about a wide range of specific alternative treatments. Table 8 outlines the number of participants that provided a buccal swab who also completed the various questionnaires.

### Statistical Comparisons

Analyses were performed using SAS 9.4 (SAS Institute Inc., Cary, NC). Graphs were produced using Excel version 14.0 (Microsoft Corp, Redmond, WA).

Normal control values were based upon the established controls from the Goldenthal laboratory published previously^19^. The two-tailed 95% normative range was considered within 1.96 +/− standard deviation of the mean, meaning that abnormal values were above 97.5% or below 2.5% of the normal distribution as defined by the control mean and standard deviation for each ETC complex measured.

Abnormalities in ETC complex activity were analyzed in several ways. First, the number of values outside of the normal range was calculated for each ETC complex measured. The binomial probability was calculated to determine if the number of values outside the normal range were significantly different than expected by chance. Second, the PMS group mean complex activity values were compared to the entire control population using t-tests. Third, a comparison was performed with age-matched controls using a pair-sample t-test. Fourth, difference in the group variance was compared using F-tests.

We also compared clinical features between groups of individuals with and without ETC complex activity abnormalities as well as individuals with and without complex I hypo vs hyper activity. For frequency variables a Fisher exact test was used. For continuous variables a t-test was used.

To determine if specific mitochondrial symptoms were significant different between PMS individuals with and without ETC complex activity abnormalities, symptoms and two-way interactions between symptoms were entered into a forward stepwise logistic regression. Selected symptoms were entered into a linear discriminant analysis with cross-validation to determine the accuracy of the ability of these symptoms to differentiate the two groups. Finally, the discriminant function was used to classify symptoms reported from additional registry responders.

## Additional Information

**How to cite this article**: Frye, R. E. *et al.* Mitochondrial Dysfunction may explain symptom variation in Phelan-McDermid Syndrome. *Sci. Rep.* **6**, 19544; doi: 10.1038/srep19544 (2016).

## Figures and Tables

**Table 1:** Genes within the Phelan-McDermid Syndrome region associated with mitochondrial function.

Gene	Position	Enzyme Name and Function	Disease Conditions
ACO2	22q13.2	• Mitochondrial aconitase	• infantile cerebellar-retinal degeneration
		• Second enzymes in the tricaboxylic acid cycle	
NDUFA6	22q13.2	• Nicotinamide adenine dinucleotide-ubiquinone oxidoreductase 1 alpha subcomplex 6	
		• Subunits of electron transport chain complex I^5^	
TRMU	22q13.31	• Transfer ribonucleic acid 5-methlaminomethyl-2-thiouridylate methyltransferase	• Aminoglycoside-induced and nonsyndromic deafness^7^
		• Modification of mitochondrial transfer ribonucleic acid^6^	• Acute infantile liver failure^8^
SCO2	22q13.33	• Homolog of S. Cerevisiae	• Fatal infantile cardioencephalomyopathy^9^,^10^,^11^,^12^,^13^
		• Assembly of electron transport chain complex IV^9^	• Spontaneous abortion^12^
		• Cytochrome c oxidase deficiency^10^	• Autism^14^
TYMP	22q13.33	• Thymidine phosphorylase	• Mitochondrial deoxyribonucleic acid depletion syndrome-1
		• Deoxynucleotide metabolism^15^	• Mitochondrial neurogastrointestinal encephalopathy^10^,^16^
CPT1B	22q13.33	• Mitochondrial carnitine palmitoyltransferase	• Heterozygous deletions can result in embryonic death or fatality after cold-challenge in mice^18^
		• Transports long-chain fatty acyl-CoA from the cytoplasm into the mitochondrial^17^	

**Table 2:** Statistical analysis of enzyme activity in children with Phelan-McDermid Syndrome.

	Citrate Synthase	Complex I Activity	Complex IV Activity	Complex II + IIIActivity	Complex IIActivity	Any Abnormality
Control Mean (SD)	12.7 (5.1)	6.8 (2.0)	0.31 (0.10)	0.092 (0.03)	0.194 (0.08)	
Normal Range	2.7–22.7	2.9–10.7	0.11–0.51	0.032–0.152	0.03–0.35	
Above Normal, %, p value	**8% (4/51), 0.04**	**14% (7/51), <0.0001**	2% (1/51), 0.93	8% (3/40), 0.08	5% (2/40), 0.26	**25% (13/51), <0.0001**
Below Normal, %, p value	0% (0/51), 1.0	**29% (15/51), <0.0001**	**14% (7/51), <0.0001**	0% (0/40), 1.0	0% (0/40), 1.0	**45% (21/51), <0.0001**
Outside Normal %, p value	8% (4/51), 0.25	**43% (22/51), <0.0001**	**16% (8/51), <0.005**	8% (3/40), 0.08	5% (2/40), 0.26	**59% (30/51), <0.0001**
PMS Mean (SD)	10.7 (6.1)	5.8 (3.8)	0.23 (0.12)	0.09 (0.07)	0.18 (0.09)	
Mean Difference, t & p value	1.72, 0.09	1.42, 0.16	**5.00, <0.0001**	0.09, 0.93	0.81, 0.41	
Variance Difference, F & p value	1.50, 0.04	**3.64, <0.00001**	1.42, 0.07	**4.80, <0.00001**	1.13, 0.03	

For Phelan-McDermid Syndrome participants, significantly more enzyme activity values were outside the normal range for complex I and IV, but not complexes II or II + III. Complex IV activity was significantly lower in Phelan-McDermid Syndrome participants as compared to controls. Variance in complex I and complex II + II were significantly different between Phelan-McDermid Syndrome participants and controls.

**Table 3:** Overlap of complex abnormalities in Phelan-McDermid Syndrome participants.

Participants with Any Complex Affected				59% (30/51)		Also Complex IV	Also Citrate Synthase
		Only One Affected		77% (24/31)			
		Two Affected		19% (6/31)			
	TotalAffected	Only ComplexAffected	AlsoComplex I	AlsoComplex II	Also ComplexII + III		
Complex I	22	68% (15/22)		0% (0)	14% (3/22)	14% (3/22)	9% (2/22)
Complex II	2	100% (2/2)	0% (0)		0% (0/2)	0% (0/2)	0% (0/2)
Complex II + III	3	0% (0/3)	100% (3/3)	0% (0/3)		0% (0/3)	0% (0/3)
Complex IV	8	63% (5/8)	25% (2/8)	0% (0/8)	0% (0/8)		38% (3/8)
Citrate Synthase	4	0% (0)	50% (2/4)	0% (0)	0% (0)	75% (3/4)	

**Table 4:** Autism Symptoms for Phelan-McDermid Syndrome participants as assessed by the Autism Symptoms Questionnaire for Phelan-McDermid Syndrome participants with and without electron transport chain complex abnormalities.

	Overall(n = 31)	No ComplexAbnormalities(n = 13)	ComplexAbnormalities(n = 18)
Autism Spectrum Disorder	61% (19/31)	69% (13/31)	56% (18/31)
Social Subscale Score	2.5 (1.3)	2.1 (1.3)	2.8 (1.3)
Communication Subscale Score	3.9 (0.7)	3.5 (0.8)	4.1 (0.6)
Stereotyped Behavior Subscale Score	2.3 (1.3)	2.4 (1.3)	2.2 (1.4)
Total Score	8.7 (2.2)	8.0 (2.1)	9.1 (2.3)

Scores from the individual subscales and the total score are also presented.

**Table 5:** Deleted Genes and deletion size reported for Phelan-McDermid Syndrome participants with and without mitochondrial complex abnormalities.

	Overall (n = 27)	No Complex Abnormalities (n = 13)	Complex Abnormalities (n = 14)
Deletion Size			
Mean (SD)	3,179,075 (2,506,868)	3,124,039 (2,659,428)	3,230,180 (2,456,505)
Median [Min;Max]	2,243,806 [7,42,694,953,953]	3,020,212 [7,046,55,144,552]	2,234,989 [7,42,694,953,953]
Deletion Type			
Terminal	73% (16/22)	64% (7/11)	82% (9/11)
Interstitial	9% (2/22)	9% (1/11)	9% (2/22)
Ring	23% (5/22)	27% (3/11)	18% (2/11)
Specific Gene Deletion			
PACSIN2	4% (1/27)	0% (0/13)	7% (1/14)
TTLL1	7% (2/27)	0% (0/13)	14% (2/14)
BIK	7% (2/27)	0% (0/13)	14% (2/14)
MCAT	7% (2/27)	0% (0/13)	14% (2/14)
TSPO	7% (2/27)	0% (0/13)	14% (2/14)
SCUBE1	7% (2/27)	0% (0/13)	14% (2/14)
MPPED1	7% (2/27)	0% (0/13)	14% (2/14)
SULT4A1	11% (3/27)	8% (1/13)	14% (2/14)
PNPLA5	11% (3/27)	8% (1/13)	14% (2/14)
PNPLA3	11% (3/27)	8% (1/13)	14% (2/14)
SAMM50	15% (4/27)	15% (2/13)	14% (2/14)
PARVB	15% (4/27)	15% (2/13)	14% (2/14)
PARVG	15% (4/27)	15% (2/13)	14% (2/14)
PRR5	15% (4/27)	15% (2/13)	14% (2/14)
ARHGAP8	15% (4/27)	15% (2/13)	14% (2/14)
NUP50	15% (4/27)	15% (2/13)	14% (2/14)
UPK3A	15% (4/27)	15% (2/13)	14% (2/14)
FBLN1	19% (5/29)	23% (3/13)	14% (2/14)
ATXN10	33% (9/29)	38% (5/13)	29% (4/14)
WNT7B	33% (9/29)	38% (5/13)	29% (4/14)
MIRLET7A3	33% (9/29)	38% (5/13)	29% (4/14)
MIRLET7B	33% (9/29)	38% (5/13)	29% (4/14)
PPARA	33% (9/29)	38% (5/13)	29% (4/14)
PKDREJ	33% (9/29)	38% (5/13)	29% (4/14)
TRMU	37% (10/27)	46% (6/13)	29% (4/14)
CELSR1	41% (11/27)	54% (7/13)	29% (4/14)
GRAMD4	41% (11/27)	54% (7/13)	29% (4/14)
CERK	44% (12/27)	54% (7/13)	36% (5/14)
BRL	81% (22/27)	69% (9/13)	93% (13/14)
ZBED4	81% (22/27)	69% (9/13)	93% (13/14)
AKG12	81% (22/27)	69% (9/13)	93% (13/14)
PIM3	81% (22/27)	69% (9/13)	93% (13/14)
IL17REL	81% (22/27)	69% (9/13)	93% (13/14)
MLC1	81% (22/27)	69% (9/13)	93% (13/14)
MOV10L1	81% (22/27)	69% (9/13)	93% (13/14)
SELO	81% (22/27)	69% (9/13)	93% (13/14)
TUBGCP6	81% (22/27)	69% (9/13)	93% (13/14)
MAPK12	81% (22/27)	69% (9/13)	93% (13/14)
PLXNB2	81% (22/27)	69% (9/13)	93% (13/14)
SAPS2	85% (23/27)	77% (10/13)	93% (13/14)
SBF1	85% (23/27)	77% (10/13)	93% (13/14)
NCAPH2	85% (23/27)	77% (10/13)	93% (13/14)
SCO2	85% (23/27)	77% (10/13)	93% (13/14)
TYMP	85% (23/27)	77% (10/13)	93% (13/14)
CPT1B	85% (23/27)	77% (10/13)	93% (13/14)
CHKB	85% (23/27)	77% (10/13)	93% (13/14)
MAPK8IP2	85% (23/27)	77% (10/13)	93% (13/14)
ARSA	85% (23/27)	77% (10/13)	93% (13/14)
SHANK3	93% (25/27)	92% (12/13)	93% (13/14)
ACR	78% (21/27)	77% (10/13)	79% (11/14)
RABL2B	63% (18/27)	62% (8/13)	64% (10/14)

**Table 6:** Prevalence of Medical Conditions and Symptoms*.

Symptoms	N	Overall	NormalMitochondrialFunction	AbnormalMitochondrialFunction
Infectious				
Ear Infections^1^	28	79% (22/28)	83% (10/12)	75% (12/16)
Sinusitis^1^	29	31% (9/29)	23% (3/13)	38% (6/16)
Throat Infections^1^	29	14% (4/29)	15% (2/13)	13% (2/16)
Immune Problem^2^	30	13% (4/30)	15% (2/13)	12% (2/17)
Allergic				
Asthma^2^	30	40% (12/30)	46% (6/13)	35% (6/17)
Allergies^1^	29	3% (1/29)	8% (1/13)	0% (0/17)
Gastrointestinal problems				
Vomiting^1^	30	20% (6/30)	23% (3/13)	18% (3/17)
Gastroesophageal Reflux^1^	29	35% (10/29)	23% (3/13)	43% (7/16)
Diarrhea^1^	30	30% (9/30)	25% (3/12)	33% (6/18)
Constipation^1^	30	57% (17/30)	54% (7/13)	59% (10/17)
Abdominal pain^1^	25	28% (7/25)	25% (3/12)	31% (4/13)
Feeding Problems^1^	31	39% (12/31)	15% (2/13)	56% (10/18)
Growth Problems				
Failure to Thrive^2^	31	32% (10/31)	15% (2/13)	44% (8/18)
Short Stature^2^	30	30% (9/30)	23% (3/13)	35% (6/17)
Tall Stature^2^	30	33% (10/30)	46% (6/13)	24% (4/17)
Obese^2^	30	20% (6/30)	23% (3/13)	18% (3/17)
Microcephaly^2^	30	23% (7/30)	23% (3/13)	24% (4/17)
Macrocephaly^2^	29	21% (6/29)	33% (4/12)	12% (2/17)
Constitutional				
Heat Intolerance^2^	29	76% (22/29)	64% (7/11)	83% (15/18)
Lack of Sweating^2^	31	68% (21/31)	69% (9/13)	67% (12/18)
Exercise Intolerance^2^	30	53% (16/30)	38% (5/13)	65% (11/17)
Fatigue^2^	31	19% (6/31)	8% (1/13)	28% (5/18)
Musculoskeletal				
Weakness^1^	30	20% (6/30)	15% (2/13)	24% (4/17)
Bone or Joint Problem^2^	30	10% (3/30)	15% (2/13)	19% (3/16)
Other Health Problems				
Heart Condition^2^	30	17% (5/30)	8% (1/13)	24% (4/17)
Urogenital Problem^2^	30	33% (10/30)	31% (4/13)	35% (6/17)
Skin Problem^2^	28	7% (2/28)	8% (1/13)	18% (3/17)
Thyroid Problems^2^	28	7% (2/28)	8% (1/12)	6% (1/16)
Dental problems^1^	30	17% (5/30)	15% (2/13)	18% (3/17)
Neurosensory				
Vision Problems^1^	29	28% (8/29)	25% (3/12)	29% (5/17)
Hearing Problems^1^	30	10% (3/30)	15% (2/13)	6% (1/17)
Headache^1^	14	14% (2/14)	14% (1/7)	14% (1/7)
Seizure^2^	29	48% (14/29)	42% (5/12)	53% (9/17)
Tics^2^	29	31% (9/29)	46% (6/13)	19% (3/16)
Strabismus^2^	31	26% (8/31)	23% (3/13)	28% (5/18)
Psychiatric Problems				
Bipolar Disorder^2^	24	8% (2/24)	18% (2/11)	0% (0/13)
Anxiety Disorder^2^	24	25% (6/24)	18% (2/11)	31% (4/13)
Obsessive-Compulsive Disorder^2^	29	38% (11/29)	38% (5/13)	38% (6/16)
Attention Deficit Hyperactivity DO^2^	30	47% (14/30)	38% (5/13)	53% (9/17)
Regression				
Regression Reported	30	74% (22/30)	71% (10/14)	75% (12/16)
Loss of Language	21	73% (16/22)	70% (7/10)	75% (9/12)
Loss of Social Skills	21	45% (10/22)	30% (3/10)	58% (7/12)
Loss of Gross Motor Skills	21	32% (7/22)	30% (3/10)	33% (4/12)
Loss of Fine Motor Skills	21	32% (7/22)	10% (1/10)	50% (6/12)
Proximal Trigger	21	45% (10/22)	30% (3/10)	58% (7/12)
ASD Regression Typical Age	21	32% (7/22)	30% (3/10)	33% (4/12)
Mean (SD) Age at Regression (in Months)	21	62 (38)	66 (40)	59 (38)
Multiple Regressions	21	45% (10/22)	50% (5/10)	42% (5/12)
Vanderbilt Questionnaires				
Attention Problems	30	27% (8/30)	50% (6/12)	11% (2/18)
Hyperactivity Problems	30	20% (6/30)	33% (4/12)	11% (2/18)
Oppositional Defiant Disorder	30	10% (3/30)	25% (3/12)	0% (0/18)
Conduct Disorder	30	0% (0/30)	0% (0/12)	0% (0/18)
Anxiety Depression	30	0% (0/30)	0% (0/12)	0% (0/18)
Sleep Questionnaire				
Amount of Sleep				
Weekday Sleep (Minutes/Night)	30	600 (60)	580 (54)	615 (61)
Weekend Sleep (Minutes/Night)	30	604 (65)	568 (45)	631 (65)
Sleep Quality				
Good	30	33% (10/30)	36% (4/12)	33% (6/18)
Mildly Abnormal	30	53% (16/30)	58% (7/12)	50% (9/18)
Severely Abnormal	30	13% (4/30)	8% (1/12)	17% (/318)
Sleep Component (%Poor)				
Bedtime Resistance	30	7% (2/30)	0% (0/12)	11% (2/18)
Sleep Onset Delay	30	7% (2/30)	8% (1/12)	6% (1/18)
Sleep Duration	30	17% (5/30)	17% (2/12)	17% (3/18)
Sleep Anxiety	30	10% (3/30)	17% (2/12)	6% (1/18)
Night Waking	30	20% (6/30)	17% (2/12)	22% (4/18)
Parasomnias	30	33% (10/30)	25% (3/12)	39% (7/18)
Sleep Disordered Breathing	30	10% (3/30)	17% (2/12)	6% (1/18)
Daytime Sleepiness	30	7% (2/30)	8% (1/12)	6% (1/18)
Alternative Therapies				
*Special Diets*				
Atkin’s Diet	31	0% (0/31)	0% (0/13)	0% (0/18)
Casein-Free Diet	32	13% (4/32)	14% (2/14)	9% (2/18)
Elemental Diet	31	0% (0/31)	0% (0/13)	0% (0/18)
Gluten-Free Diet	32	13% (4/32)	21% (3/14)	6% (1/18)
Ketogenic Diet	31	0% (0/31)	0% (0/13)	0% (0/18)
No Processed Sugar	31	3% (1/31)	8% (1/13)	0% (0/18)
Specific Carbohydrate Diet	31	0% (0/31)	0% (0/13)	0% (0/18)
*Gastrointestinal Therapies*				
Digestive enzymes	31	10% (3/31)	8% (1/13)	11% (2/18)
Probiotic	32	34% (11/32)	36% (5/14)	33% (6/18)
*Vitamin Supplementations*				
Amino Acids	31	0% (0/31)	0% (0/13)	0% (0/18)
B12	32	3% (1/32)	7% (1/14)	0% (0/18)
Carnitine/Acetyl-L-Carnitine	31	7% (2/31)	0% (0/13)	11% (2/18)
Essential Fatty Acids	31	7% (2/31)	7% (1/13)	6% (1/18)
Folinic Acid	31	0% (0/31)	0% (0/13)	0% (0/18)
Glutathione	31	0% (0/31)	0% (0/13)	0% (0/18)
High-dose B Vitamins	31	7% (2/31)	15% (2/13)	0% (0/18)
N-Acetyl-cysteine	31	0% (0/31)	0% (0/13)	0% (0/18)
*Other Therapies*				
Chiropractic	32	6% (2/32)	14% (2/14)	0% (0/18)
Homeopathy	31	7% (2/31)	15% (2/13)	0% (0/18)

*Questions not included in Table 4 because no positive answers were obtained include congenital heart disease, heart failure, growth hormone deficiency, syncopy, ophthalmoplegia, cerebral palsy, depression and schizophrenia.

^1^Current or Previous with Moderate or Severe Severity.

^2^Current or Previous with Any Severity.

**Table 7:** Finding associated with complex I under and over activity.

	Complex IUnderactivity	Complex IOveractivity
Development		
ASD	44% (4/9)	75% (3/4)
Regression	63% (5/8)	100% (4/4)
Loss of Language	80% (4/5)	75% (3/4)
Loss of Social Skills	40% (2/5)	100% (4/4)
Loss of Gross Motor Skills	60% (3/5)	0% (0/4)
Loss of Fine Motor Skills	80% (4/5)	25% (1/4)
Proximal Trigger	80% (4/5)	0% (0/4)
ASD Regression Typical Age	20% (1/5)	75% (3/4)
Mean (SD) Age at Regression	63 m (29 m)	27 m (16 m)
Multiple Regressions	60% (3/5)	25% (1/4)
Gene Deletion		
TRMU	38% (3/8)	0% (0/3)
SCO2/TYMP/CPT1B	88% (7/8)	100% (3/3)
SHANK3	100% (8/8)	66% (2/3)
Gastrointestinal problems		
Vomiting^1^	0% (0/8)	0% (0/4)
Gastroesophageal Reflux^1^	43% (3/7)	25% (1/4)
Diarrhea^1^	33% (3/9)	50% (2/4)
Constipation^1^	88% (7/8)	25% (1/4)
Abdominal pain^1^	50% (3/6)	0% (0/4)
Feeding Problems^1^	56% (5/9)	50% (2/4)
Growth Problems		
Failure to Thrive^2^	44% (4/9)	25% (1/4)
Short Stature^2^	44% (4/9)	0% (0/4)
Tall Stature^2^	22% (2/9)	25% (1/4)
Obese^2^	22% (2/9)	0% (0/4)
Microcephaly^2^	22% (2/9)	0% (0/4)
Macrocephaly^2^	22% (2/9)	25% (1/4)
Constitutional		
Heat Intolerance^2^	89% (8/9)	75% (3/4)
Lack of Sweating^2^	67% (6/9)	50% (2/4)
Exercise Intolerance^2^	63% (5/8)	25% (1/4)
Fatigue^2^	33% (3/9)	0% (0/4)
Weakness^1^	25% (2/8)	0% (0/4)
Neurosensory		
Vision Problems^1^	38% (3/8)	0% (0/4)
Hearing Problems^1^	0% (0/9)	0% (0/4)
Headache^1^	0% (0/3)	50% (2/4)
Seizure^2^	63% (5/8)	25% (1/4)
Tics^2^	11% (1/9)	25% (1/4)
Strabismus^2^	44% (4/9)	0% (0/4)
Psychiatric Problems		
Bipolar Disorder^2^	0% (0/5)	0% (0/4)
Anxiety Disorder^2^	29% (2/7)	50% (2/4)
Obsessive-Compulsive Disorder^2^	44% (4/9)	25% (1/4)
Attention Deficit Hyperactivity DO^2^	67% (6/9)	25% (1/4)

^1^Current or Previous with Moderate or Severe Severity.

^2^Current or Previous with Any Severity.

**Table 8:** Percentage of participants with completed information in the Phelan-McDermid Syndrome Foundation Registry.

Deletion Size and Position	53% (27/51)
Autism Symptoms Questionnaire	61% (31/51)
Regression Questionnaire	59% (30/51)
Medical Questionnaire	61% (31/51)
Behavioral Questionnaire	61% (31/51)
Sleep Questionnaire	59% (30/51)
Alternative Medications and Treatments	63% (32/51)
